# A comparison of intrauterine hemopoietic cell transplantation and lentiviral gene transfer for the correction of severe β-thalassemia in a HbbTh3/+ murine model

**DOI:** 10.1016/j.exphem.2018.03.006

**Published:** 2018-06

**Authors:** Niraja M. Dighe, Kang Wei Tan, Lay Geok Tan, Steven S.W. Shaw, Suzanne M.K. Buckley, Dedy Sandikin, Nuryanti Johana, Yi-Wan Tan, Arijit Biswas, Mahesh Choolani, Simon N. Waddington, Michael N. Antoniou, Jerry K.Y. Chan, Citra N.Z. Mattar

**Affiliations:** a Experimental Fetal Medicine Group, Department of Obstetrics and Gynaecology, Yong Loo Lin School of Medicine, National University of Singapore, 119228 Singapore, Singapore; b College of Medicine, Chang Gung University, 33302 Taoyuan, Taiwan, China; c Prenatal Cell and Gene Therapy Group, Institute for Women's Health, University College London, WC1E 6AU London, United Kingdom; d Gene Transfer Technology Group, Institute for Women's Health, University College London, WC1E 6AU London, United Kingdom; e Department of Reproductive Medicine, KK Women's and Children's Hospital, 229899 Singapore, Singapore; f MRC Antiviral Gene Therapy Research Unit, Faculty of Health Sciences, University of the Witwatersrand, Johannesburg, South Africa; g Gene Expression and Therapy Group, King's College London, Faculty of Life Sciences and Medicine, Department of Medical and Molecular Genetics, Guy's Hospital, SE1 9RT London, United Kingdom; h Cancer and Stem Cell Program, Duke-NUS Graduate Medical School, 169857 Singapore, Singapore

## Abstract

Major hemoglobinopathies place tremendous strain on global resources. Intrauterine hemopoietic cell transplantation (IUHCT) and gene transfer (IUGT) can potentially reduce perinatal morbidities with greater efficacy than postnatal therapy alone. We performed both procedures in the thalassemic HbbTh3/+ mouse. Intraperitoneal delivery of co-isogenic cells at embryonic days13-14 produced dose-dependent chimerism. High-dose adult bone marrow (BM) cells maintained 0.2–3.1% chimerism over ~24 weeks and treated heterozygotes (HET) demonstrated higher chimerism than wild-type (WT) pups (1.6% vs. 0.7%). Fetalliver (FL) cells produced higher chimerism than BM when transplanted at thesame doses, maintaining 1.8–2.4% chimerism over ~32 weeks. We boosted transplanted mice postnatally with BM cells after busulfan conditioning. Engraftment was maintained at >1% only in chimeras. IUHCT-treated nonchimeras and non-IUHCT mice showed microchimerism or no chimerism. Improved engraftment was observed with a higher initial chimerism, in HET mice and with the addition of fludarabine. Chimeric HET mice expressed 2.2–15.1% engraftment with eventual decline at 24 weeks (vs. <1% in nonchimeras) and demonstrated improved hematological indices and smaller spleens compared with untreated HETmice. Intravenous delivery of GLOBE lentiviral-vector expressing human β-globin (HBB) resulted in a vector concentration of 0.001–0.6 copies/cell. Most hematological indices were higher in treated than untreated HET mice, including hemoglobin and mean corpuscular volume, but were still lower than in WT. Therefore, direct IUGT and IUHCT strategies can be used to achieve hematological improvement but require further dose optimization. IUHCT will be useful combined with postnatal transplantation to further enhance engraftment.

## Highlights

• The HbbTh3/+ mouse is a good model of severe thalassemia for in utero therapy.
• In utero and postnatal transplantation with immunosuppression resulted in better chimerism.
• In utero gene therapy produced partial hematological correction but not full rescue.
• Both strategies need further optimization to overcome the hostile microenvironment.

The hemoglobinopathies are the most prevalent monogenetic disorders and generate substantial medical and socioeconomic burden worldwide [1]. α-Thalassemia major is perinatally lethal and necessitates effective intrauterine intervention to avoid the complications of chronic severe hypoxia evident in transfusion-dependent survivors [2]. β-Thalassemia major and sickle cell disease (SCD) demand substantial resources to prevent permanent organ failure [3]. Much of the disease burden arises from suboptimal treatment [4]. Curative postnatal allogeneic hemopoietic stem cell transplantation (HSCT) is available to one-third of individuals with thalassemia and requires bone marrow (BM) conditioning, risking well-documented complications [5]. Due to the projected expansion of at-risk populations, there is an urgent need to formulate an early intervention strategy that is effective and safe [6]. Although α-thalassemia major clearly requires an intrauterine remedy given the early fatality, fetal treatment of β-hemoglobinopathies is debatable as clinical manifestations only arise in infancy. However, acknowledging the risks of conventional treatment and the therapeutic advantage of youth, a strong argument can be made for fetal therapy in which the goal is reduction of disease burden 7, 8. Potential benefits of intrauterine cell and gene therapy for these and other genetic disorders are widely described 9, 10. Notable advantages of intrauterine hemopoietic cell transplantation (IUHCT) include the high donor cell:fetal mass ratio (dose-dependent response), immune naiveté (donor cell tolerance), and diminished host competition for available hemopoietic niches [11]. Advantages of intrauterine gene transfer (IUGT) include the greater transducibility of fetal target cells and lower risk of immune-mediated clearance [10]. Potential correction of these conditions well before irreversible end-organ damage and avoidance of treatment-related morbidity underscores the expectation that intrauterine therapies will benefit both α- and β-thalassemia major, similar to treatment of congenital immunodeficiency syndromes and osteogenesis imperfecta 2, 12.

Despite its promise, IUHCT has been largely disappointing in most monogenetic conditions due to host immune and competitive barriers [13]. In mice, achieving sustained engraftment within a competent host immune system requires a minimum initial donor cell chimerism of 1.8% [14]. Although higher engraftment has been achieved in animal models, therapeutic engraftment has been difficult to replicate in humans [15]. The unique microenvironment in the BM of thalassemic individuals and the lack of a competitive advantage for donor cells suggests that a strategy more complex than a single IUHCT may be needed to reach therapeutic effect [16], such as transplanting high-dose maternal donor cells within the optimal gestational window and T-cell manipulation of the donor inoculum 17, 18, 19. The alternative approach of in vivo IUGT has been utilised in a murine α-thalassemia model to achieve erythroid-specific α-globin expression lasting seven months [20]. In adult individuals with β-thalassemia, ex vivo gene therapy has met with reasonable success, but this approach is impractical in the fetus because it necessitates multiple invasive procedures [21]. IUGT may present an effective way to target fetal hemopoietic progenitors and has demonstrated success in treating other models of monogenetic disease [9]. HIV-1-based integrating lentiviral vectors (LVs) are valuable in the treatment of hemoglobinopathies because they transduce quiescent hemopoietic stem cells (HSCs), are less mutagenic than γ-oncoretroviruses, and are becoming safer and more efficient for clinical use through improved design 21, 22. To study and compare the outcomes of IUHCT and IUGT, we used the HbbTh3/+ murine model in which surviving HET mice clinically represent severe β-thalassemia intermedia and nonsurviving homozygotes represent α-thalassemia major [23]. We examined the additive effect of postnatal transplantation after IUHCT and the efficacy of a single intrauterine injection of LV-MA821 (GLOBE) expressing a human β -globin (*HBB*) transgene [24].

## Methods

### Animal experiments

Animal experiments were performed at the National University of Singapore (NUS), an Association for Assessment and Accreditation of Laboratory Animal Care International-accredited institution and followed guidelines described in the National Institutes of Health's *Guide for the Care and Use of Laboratory Animals* under the NUS Institutional Animal Care and Use Committee and the Office of Safety, Health and Environment. B6.129P2-Hbb-b1^tm1Unc^Hbb-b2^tm1Unc^/J mice (HbbTh3/+) and C57BL/6.CD45.1 (B6) mice were purchased from The Jackson Laboratory (Bar Harbor, ME) and Crl:CD1[ICR] females (CD1) were purchased from Charles River Laboratories (Wilmington, MA). C57BL/6-Tg[UBC-GFP]30Scha/J.CD45.2 mice (B6-GFP) mice used as BM donors at 6–8 weeks were kindly donated by F. Ginhoux (Singapore Immunology Network, A*STAR, Singapore). HbbTh3/+ males and HbbTh3/+ or B6 females were mated for IUHCT performed intraperitoneally (IP) at embryonic day 13 (E13) to E14 and HbbTh3/+ males and CD1 females for IUGT performed intravenously (IV) at E15–E16. The day of plug observation was designated E0.5 (Supplementary Figure E1, online only, available at www.exphem.org). Animals were sacrificed with inhalational CO_2_ or cervical dislocation for terminal harvests.

### Murine BM and FL donor cell preparation

BM cells and FL donor cells were harvested from long bones of B6-GFP adults and E13–E14 fetuses, respectively. Cells collected in phosphate-buffered saline (PBS; Invitrogen, Grand Island, NY, USA) containing 2 mmol/L ethylenediaminetetraacetic acid (EDTA, Sigma-Aldrich) at pH 7.2 were processed into single-cell suspensions by passage through a 22-gauge needle and centrifuged over 15 mL of Ficoll-Histopaque 1077 (Sigma-Aldrich) as described previously [25]. Mononuclear cells (MNCs) were harvested from the interphase layer, washed in PBS, frozen, and thawed in batches for IUHCT. Donor MNCs were consistently >95% for green fluorescent protein (GFP), ~70% negative for lineage markers (Lin^–^), and ~20-40% cKit^+^ and Sca1^+^, so we used the whole MNC component without further enrichment to prevent potential loss of proliferating long-term repopulating HSCs [26]. Viable MNCs confirmed by trypan blue exclusion were T-cell depleted with fluorescein isothiocyanate (FITC) anti-mouse CD3-antibody, incubated with anti-FITC microbeads, and passaged through magnetic columns (Mouse CD3 Depletion Kit, Miltenyi Biotec, Singapore) [27]. Flow cytometry (fluorescence-activated cell sorting, FACS) confirmed a CD3^+^ component <0.5% of the final inoculum. Final cell concentrations were prepared according to live cell counts. CD26 inhibition was performed with Diprotin A (Peptides International, Louisville, KY) before transplantation as described previously [28].

### LV preparation

GLOBE is a self-inactivating HIV-based LV encoding mini-*HBB* and linked to the HS2 and HS3 elements of the *HBB* locus control region [29]. LV stocks were generated by triple plasmid cotransfection of human embryonic kidney 293 (HEK293T) cells using the packaging plasmid pCMVδR8.2 (9.75 µg), the envelope plasmid pMD2.G (5.25 µg), and the transfer vector plasmid MA821 (15 µg for transfection of a single 75 cm^2^ flask) with a Calcium Phosphate Transfection Kit (Invitrogen, USA) as described previously 24, 30. Briefly, medium was collected after 48 and 72 hours of transfection, vector particles were concentrated by centrifugation (90,000 *g*, 140 minutes, 4°C), pellets were resuspended in sterile PBS with 1% bovine serum albumin, and titers were quantified by quantitative PCR (qPCR) using forward primer 5′-TGAAAGCGAAAGGGAAACCA-3′ and reverse primer 5′-CCGTGCGCGCTTCAG- 3′ specific for the rev-response element (RRE) region [24].

### Intrauterine cell and vector administration

All procedures were performed under isoflurane anesthesia with dams given caprofen and enrofloxacin (at 0.1 mL/10 g body weight) before midline laparotomies at which uterine horns were exteriorized and injections administered with a 34-gauge Hamilton needle (Bonaduz, Switzerland) under a stereomicroscope. For IUHCT, murine BM GFP^+^ MNCs (B6-GFP) were delivered IP in 10 µL, at 2E+6 (low-dose, BM^LD^) or 5E+6 (high-dose, BM^HD^) cells/fetus. FL cells were injected IP at 2E+6 (FL^LD^) or 5E+6 (FL^HD^) cells/fetus. For IUGT, a 20 µL suspension of GLOBE was administered IV via the vitelline peripheral yolk sac vessel at 5E+6 transforming units (TU)/fetus 31, 32. We tried to inject all fetuses in each litter. The maternal abdomen was closed in two layers with absorbable polyglactin sutures. Dams recovered in clean, warm cages and were kept in a quiet environment until they littered. Pups were cross-fostered on coparturient CD1 dams and genotyped (see supplementary data, online only, available at www.exphem.org). Postnatal transplantations were performed via tail-vein injections after IP busulfan and with CD26 inhibition of adult BM donor cells 25, 28; some animals also received IV fludarabine (see supplementary data).

### Surveillance and terminal harvest

Blood samples collected via tail venipuncture were analyzed for hemoglobin concentration (Hb), red blood cell (RBC) counts, RBC indices, and hematocrit (Hct) on a HemaVet 950 automated blood cell analyzer (Drew Scientific, FL). Blood smears were stained with May–Grunwald–Giemsa. B6-GFP chimerism was assessed in the peripheral blood MNCs of HET and WT recipients for 24–32 weeks of age by FACS for GFP and anti-mouse CD45.2 (conjugated to allophycocyanin) and calculated as the percentage of GFP^+^ cells as a fraction of the total CD45.2 MNC population (all antibodies were from BioLegend, Singapore). A total of 50,000–100,000 events were analyzed for FACS. Vector biodistribution was assessed with 15 ng of genomic DNA serially extracted from nucleated peripheral blood cells and from other tissues at terminal harvest [31] in a qPCR using standard late reverse transcript (LRT) primers specific for the RRE region complementary to the vector backbone (forward primer 5′-TGAAAGCGAAAGGGAAACCA-3′, reverse primer 5′-CCGTGCGCGCTTCAG-3′) with mouse *ACTB* as a loading control (forward primer 5′- GGTGCTAAGAAGGCTGTT-3′ and reverse primer 5′- GGATACCTCTCTTGCTCTG-3′) for estimation of average vector copy number (VCN) per cell.

### Statistical analysis

Parametric data are shown as mean ± standard deviation (SD) and were analyzed using one-way or two-way analysis of variance and unpaired *t* tests. Nonparametric data were analyzed using the Mann–Whitney test. *p* ≤ 0.05 was considered significant. Analyses were performed on GraphPad Prism version 6.04 software (La Jolla, CA).

## Results

### IUHCT with adult BM and FL donor cells

**Figure 1:**
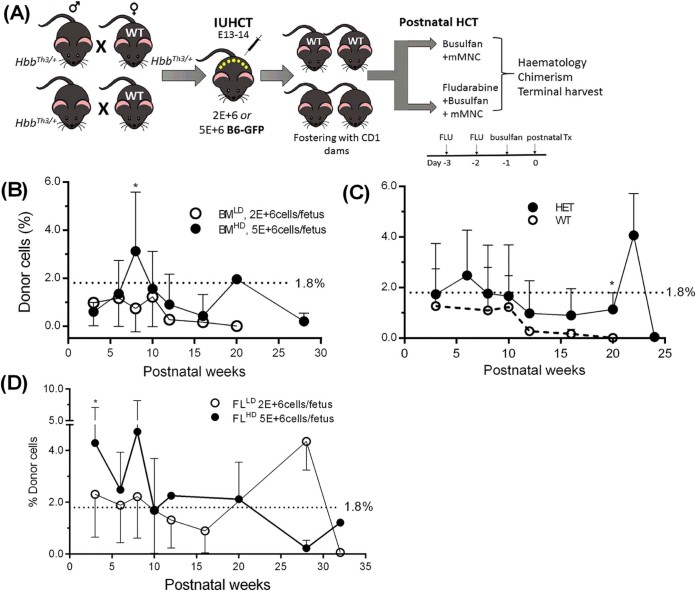
Murine adult BM and FL mononuclear cells transplanted into HbbTh3 murine fetuses. **(A)** BM^LD^ (2E+6) or BM^HD^ (5E+6) B6-GFP donor cells were administered to E13–E14 pups (HbbTh3/+ males × HbbTh3/+ or B6 females). **(B)** This produced overall chimerism of 0.6 ± 0.5% vs. 1.3 ± 0.9%, respectively (*p* = 0.06), significant at 8 weeks (0.7 ± 0.9% vs. 3.1 ± 2.5%, *p* = 0.001). **(C)** HET mice had higher chimerism compared with WT after BM^LD^ treatment (1.6 ± 0.4% vs. 0.7 ± 0.2, *p* = 0.05) and maintained low chimerism of 0.9–4.1% until 24 weeks, whereas WT chimerism (0.2–1.7%) was lost by 20 weeks. **(D)** FL^LD^ demonstrated 0.9–4.4% chimerism until 32 weeks; FL^HD^ showed 0.2–4.7% chimerism (>1% at 32 weeks). Mean chimerism was similar between the groups (2.4 ± 1.5% FL^HD^ vs. 1.8 ± 1.2% FL^LD^, *p* = 0.45). There were no surviving HET FL-IUHCT pups. *p <* *0.05.

To investigate the ability to rescue HbbTh3/+ and β^0^/β^0^ homozygotes, we performed IP-IUHCT with co-isogenic B6-GFP cells and compared outcomes with FL cells at the same doses (Fig. 1A). There were no surviving homozygotes. BM^HD^ (*n* = 10) demonstrated higher overall chimerism than BM^LD^ (*n* = 14) over 20 weeks (1.3 ± 0.9% vs. 0.6 ± 0.5%, *p* = 0.06) with chimerism ranging from 0.2% to 3.1% and 0.01% to 1.2%, respectively. Differences between both groups were significant at 8 weeks (BM^HD^ 3.1 ± 2.5 vs. 0.7 ± 0.9 BM^LD^, *p* = 0.001, Fig. 1B). HET mice (*n* = 9) showed higher chimerism compared with WT (*n* = 6) after BM^LD^ treatment (1.6 ± 0.4% vs. 0.7 ± 0.2, *p* = 0.05); differences were significant at 20 weeks (1.1% vs. 0.01%, *p* = 0.006). HET mice maintained low chimerism of 0.9–4.1% until 24 weeks, whereas WT (initially 0.2–1.7%) lost engraftment by 20 weeks (Fig. 1C). Because all surviving FL-IUHCT pups were WT, comparisons were made with BM-IUHCT WT mice injected in the same batch. FL^LD^ (*n* = 8) demonstrated chimerism of 0.9–4.4% (mean 1.8 ± 1.3%, undetectable at 32 weeks), whereas FL^HD^ (*n* = 8) showed 0.2–4.7% chimerism (mean 2.4 ± 1.5%, >1% at 32 weeks); both groups maintained stable low chimerism until ~28–32 weeks (Fig. 1D). Mean chimerism was similar between the groups (2.4 ± 1.5% FL^HD^ vs. 1.8 ± 1.2% FL^LD^, *p* = 0.45). Higher chimerism resulted from FL^HD^ compared with BM^HD^ (2.4 ± 1.5% vs. 1.2 ± 0.5%, *p* = 0.07); this difference was significant at 3 weeks (4.3% vs. 0.6%, *p *<* *0.05). FL^LD^ showed similar overall chimerism to BM^HD^ (1.8 ± 1.3% vs. 1.2 ± 0.6%, *p* = 0.07) and higher chimerism than BM^LD^ (1.8 ± 1.3% vs. 0.7 ± 0.5%, *p* = 0.03).

### Postnatal transplantation with busulfan and fludarabine

**Figure 2:**
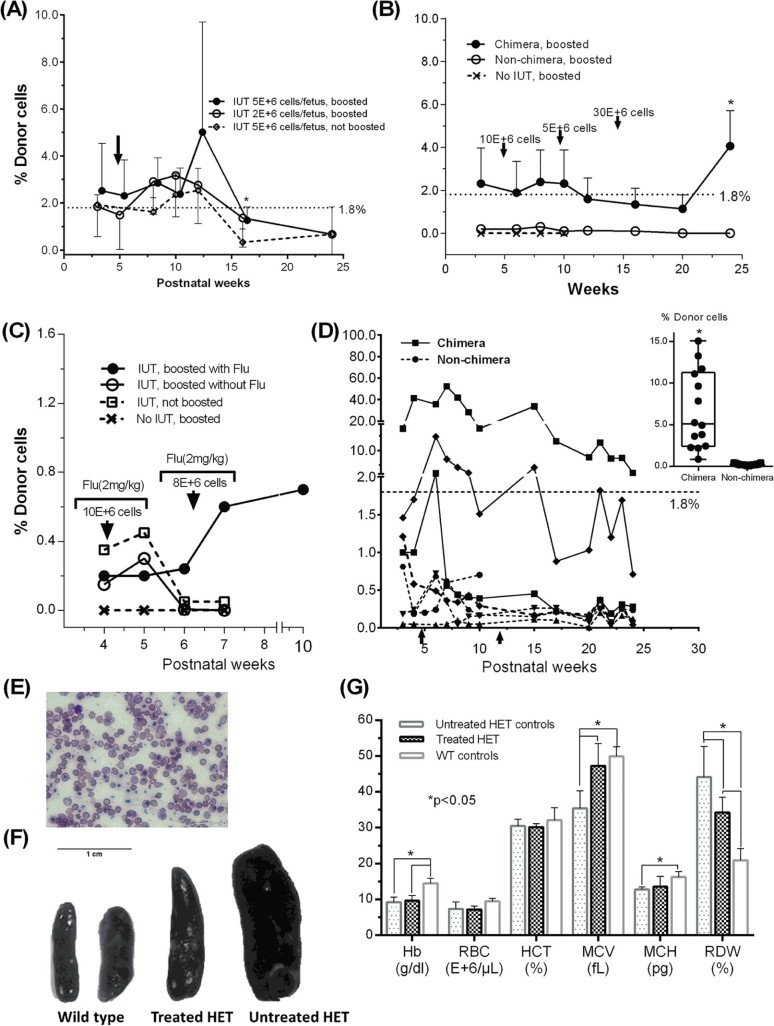
Postnatal transplantation to boost chimerism after intrauterine transplantation. Adult BM mononuclear cells were postnatally transplanted into chimeric and nonchimeric IUHCT recipients to boost engraftment. Black arrows indicate time points of postnatal transplantation. **(A)** BM^HD^ and BM^LD^ IUHCT recipients were given 5E+6 cells IV at 5 weeks. BM^LD^ produced 2.0 ± 0.9% chimerism (range 0.7–3.2%) and BM^HD^ produced 2.7 ± 1.2% chimerism (range 1.3–5.0%, *p* = 0.3). Both lost chimerism after 16–20 weeks. Unboosted BM^HD^ controls maintained 1.6 ± 0.9% chimerism. **(B)** Multiple boosts to BM^LD^ chimeras and nonchimeras with 5–30E+6 BM cells produced higher donor cell levels in chimeras (2.1 ± 0.9 vs 0.1 ± 0.1, *p *<* *0.001), which was maintained mostly >1.8% until 24 weeks. Non-IUHCT-boosted mice (*n* = 3) showed no postnatal chimerism. **(C)** Transient immunosuppression was produced with IV fludarabine in BM^LD^ recipients (<1% chimerism after IUHCT) before postnatal boosts of 10E+6 BM cells at 4 weeks and 8E+6 cells at 6 weeks. Fludarabine-treated mice maintained chimerism at 0.2–0.7% after the second boost and had higher donor cell levels than non-fludarabine-treated controls (0.4 ± 0.2% vs. 0.1 ± 0.1% respectively, *p* = 0.3). IUHCT mice with and without postnatal boost and non-IUHCT-treated pups transplanted postnatally showed minimal or no chimerism. **(D)** BM^HD^ mice were treated with fludarabine and 10E+6 cells twice at 4 and 10 weeks. Chimeras showed increased donor cell levels after each boost (overall chimerism 6.7 ± 4.6%) before eventual wastage to <1% by 24 weeks. Nonchimeras did not show improvement after either boost (inset). Two treated chimeras maintained levels of 2.3–52.4% and 1.0–14.7% and were HET by genotype. Both had ongoing hemolysis **(E)** and smaller (though still enlarged) spleens **(F)** compared with nonchimeras. **(G)** Treated HET mice showed increased MCV and reduced RDW compared with untreated HET mice (47.3 ± 6.2 fL vs. 35.4 ± 4.9 fL, and 34.3 ± 4.3% vs. 44.1 ± 8.6%, respectively, *p *<* *0.05), approaching the levels of WT. Treated HET mice had lower Hb than WT, but there were no differences in MCV and MCH (49.9 ± 2.6 fL vs. 47.3 ± 6.2 fL and 16.2 ± 1.6 pg vs. 13.6 ± 2.9 pg, respectively, *p* > 0.05). *p <* *0.05.

To counter early engraftment loss, we boosted chimeric (>1%) mice postnatally with B6-GFP adult BM cells with single and multiple doses. BM^HD^ and BM^LD^ IUHCT recipients were given 5E+6 cells IV at 5 weeks (Fig. 2A). BM^LD^ produced chimerism of 2.0 ± 0.9% (range 0.7–3.2%) similar to BM^HD^ of 2.7 ± 1.2% (1.3–5.0%, *p* = 0.3). Both lost chimerism after 16–20 weeks. Unboosted BM^HD^ controls maintained 1.6 ± 0.9% chimerism; differences between BM^HD^ and controls were significant at 16 weeks (1.3% vs. 0.3%, *p* = 0.04). We performed multiple postnatal boosts to maintain engraftment in BM^LD^ chimeras (>1%, *n* = 8) and nonchimeras (*n* = 8) using 5–30E+6 BM cells at 5, 10, and 15 postnatal weeks (Fig. 2B). Post-boost chimerism was higher in chimeras compared with boosted nonchimeras (2.1 ± 0.9 vs 0.1 ± 0.1, p < 0.001); individual time-point differences were significant at 24 weeks (4.1 vs 0.001%, *p* = 0.03). Chimerism was maintained >1% and mostly >1.8% until 24 weeks. Non-IUHCT-boosted mice (*n* = 3) showed no postnatal chimerism. To assess the effect of transient immunosuppression on sustained engraftment, a preliminary assessment of fludarabine use was performed on BM^LD^ mice, which showed <1% chimerism after IUHCT (Fig. 2C). Mice were given 10E+6 BM cells at 4 weeks and 8E+6 cells at 6 weeks (doses varied due to donor cell availability). Fludarabine treatment (*n* = 2) in addition to busulfan maintained chimerism at 0.2–0.7% after the second boost and resulted in higher donor cell levels than busulfan alone (*n* = 2, 0.4 ± 0.2% vs. 0.1 ± 0.1%, respectively, *p* = 0.3). No differences in chimerism were observed between IUHCT mice with and without postnatal boost in this group. Non-IUHCT-treated pups transplanted postnatally showed no chimerism.

We then boosted BM^HD^ mice with fludarabine and busulfan twice at 4 weeks and 10 weeks to determine engraftment duration, each dose being 10E+6 cells (Fig. 2D). IUHCT-treated chimeras (*n* = 4) demonstrated 5.2 ± 8.1% donor cell levels before postnatal boost, which peaked at 15.1 ± 25.1% and decreased to 4.9 ± 8.4% after the first boost. The second produced a further increase to 9.6 ± 16.2% before eventual wastage to <1% by 24 weeks. Nonchimeras (*n* = 4) did not show improvement after either boost, with levels remaining at 0.1–0.4%, significantly lower than chimeras (0.2 ± 0.1% vs. 6.7 ± 4.6, *p *<* *0.0001; Fig. 2D, inset). The highest expressers maintained levels of 2.3–52.4% and 1.0–14.7% and were HET by genotype. Both had smaller (though still enlarged) spleens compared with nonchimeras and untreated HET mice and ongoing hemolysis (Figs. 2E and 2F). Treated HET mice showed increased mean corpuscular volume (MCV) and reduced RBC distribution width (RDW) compared with untreated HET mice (47.3 ± 6.2 fL vs. 35.4 ± 4.9 fL and 34.3 ± 4.3% vs. 44.1 ± 8.6%, respectively, *p *<* *0.05), approaching the levels of WT (Fig. 2G). Although treated HET mice still had lower Hb than WT mice, there were no differences in MCV and mean corpuscular Hb (MCH) (49.9 ± 2.6 fL vs. 47.3 ± 6.2 fL and 16.2 ± 1.6 pg vs. 13.6 ± 2.9 pg, respectively, *p* > 0.05).

### IUGT with GLOBE

**Figure 3:**
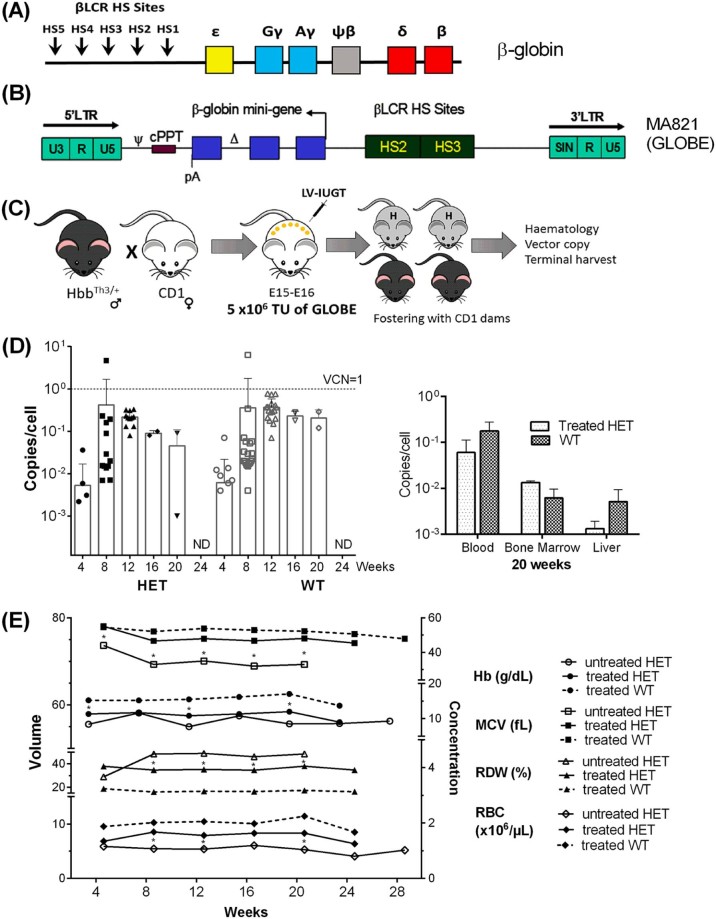
Intrauterine injection of lentiviral vectors expressing murine B globin in HbbTh3 murine fetuses. To examine the effectiveness of IUGT, we administered IV injections of GLOBE **(A)**, a lentiviral vector constructed to express the human β-globin minigene in the erythropoietic lineage, driven by the β-locus control region (β-LCR, comprising HS2/HS3). **(B)** Pups were injected at E15–E16 with a LV dose of 5E+6 TU in 20 µL via the perivitelline vein. **(C)** VCN in treated HET and WT mice were similar all time points (means 0.1 and 0.2 copies/cell respectively, *p* = 0.5); both groups demonstrated an increasing VCN that peaked at 8 weeks (mean ~0.4 ± 1.4 copies/cell) and was not detectable (ND) by 24 weeks. **(D)** There were no differences in low-level VCN in blood, BM, and liver at 20 weeks. **(E)** Hematological parameters showed an overall improvement in treated versus untreated HET mice: Hb (11.0 ± 1.1g% vs. 9.3 ± 1.4g%, *p* = 0.04), MCV (48.3 ± 3.5 fL vs. 35.4 ± 4.9 fL, *p* = 0.004), and RBC (7.7 ± 0.9 × 10^6^/µL vs. 5.3 ± 0.6 × 10^6^/µL, *p* = 0.001) with reduced RDW (35.8 ± 1.6% treated vs. 44.1 ± 8.6% untreated, *p* = 0.1). *p <* *0.05.

We generated GLOBE concentrates containing ~2.6E+8 TU/mL (Fig. 3A). We injected 5E+6 TU of GLOBE IV into hybrid fetuses at E15–E16 to assess in vivo IUGT (Fig. 3B). Vector biodistribution and hematological indices in GLOBE-injected mice and controls were monitored from 3 to 24 weeks postpartum. There was no difference in MNC VCN in peripheral blood between HET mice (*n* = 13, 0.1 ± 0.2 copies/cell) and WT mice (*n* = 20, 0.2 ± 0.1 copies/cell, *p* = 0.5) detectable until 20 weeks after IUGT. Monthly VCN ranged from 0.001 to 0.4 copies/cell in both groups (Fig. 3C). Terminal analyses at 20 weeks (*n* = 3) showed no VCN differences between treated HET and WT mice in the blood (0.06 ± 0.05 vs. 0.18 ± 0.09 copies/cell), BM (0.01 ± 0.01 vs. 0.006 ± 0.003 copies/cell), or liver (0.001 ± 0.001 vs. 0.005 ± 0.004 copies/cell) (Fig. 3D). Most hematological indices were higher in treated than in untreated HET mice: Hb (11.0 ± 1.1g% vs. 9.3 ± 1.4g%, *p* = 0.04), MCV (48.3 ± 3.5 fL vs. 35.4 ± 4.9 fL, *p* = 0.004) and RBC (7.7 ± 0.9E+6/µL vs. 5.3 ± 0.6E+6/µL, *p* = 0.001), with a trend toward normal RDW (35.8 ± 1.6% vs. 44.1 ± 8.6%, *p* = 0.1). Significant time point differences between treated and untreated HET mice are shown in Fig. 3E. Hematological values remained statistically lower in treated HET mice compared with WT mice.

## Discussion

We demonstrate partial improvement in the HbbTh3/+ mouse model with two clinically applicable strategies of low-dose *in vivo* GLOBE-IUGT and IUHCT with postnatal boost with fludarabine, which achieved preferential engraftment in chimeras and HET mice over nonchimeras and WT offspring. Although we did not achieve full phenotypic correction with either, we observed that most hematological indices in treated HET mice improved compared with nontreated HET mice, an effect marginally superior to IUGT despite the low VCN. IUHCT has long been limited by low-level chimerism in animal models due to steadfast engraftment barriers 33, 34, which have been challenged though manipulation of donor cells and recipient BM microenvironment.

We performed IP-IUHCT with a dose of 2–5E+6 cells/fetus, similar to studies demonstrating dose-dependent chimerism of 0–7.5% (2E+4 to 2E+6 cells/fetus IP), 10–20% (2.5E+6 cells/fetus intrahepatic, IH) and 5–20% (5E+6 cells/fetus IP) with eventual long-term decline in WT mice 14, 35, 36 and stable chimerism of 0.5–1.7% in the CLAD canine model (IP, 2–5E+8 CD34^+^ cells/kg) [37]. In thalassemia and SCD mice, however, single allogeneic IP-IUHCT of 5E+6 adult BM cells/pup resulted in lower chimerism of 1.1–4.1% over 10 months 33, 38. These data are informative of the expected chimerism resulting from a starting IP/IH dose of 2–5E+6 cells of ~5–20% in WT mice and ~1–4% in hemoglobinopathy models. Our donor cell chimerism of 2.3–4.9% at 3 postnatal weeks is comparable, but the duration of engraftment was much shorter without postnatal boost. We administered ~4–10E+9 cells/kg at E14–16 (weights 0.5–0.7 g), two log-folds higher than the recommended dose for optimal engraftment in postnatal umbilical cord blood (UCB) transplantations of ~4E+7 nucleated cells/kg, albeit with conditioning therapy [39]. The engraftment shortfall in our model reflects other physiological barriers such as the lack of space, immune rejection, and technical barriers. Although IV injections allow delivery of a much higher cell dose (20–30E+6 vs. 5E+6 IP) with significantly higher short-term engraftment [40], there is little difference in long-term engraftment between IV-IUHCT (20E+6 cells/fetus, 5–10% at 6 months 28, 40) and IH or IP-IUHCT (2.5–5E+6 cells/fetus, 5–20% 14, 35, 36). IV-IUHCT at this early gestation is technically demanding and IP or IH injections are more reproducible, an important consideration for clinical application, particularly when fetuses are targeted in early gestation [41]. Congenic IUHCT produces lower, but ultimately more stable, chimerism compared with the allogenic IUHCT, in which the majority of engrafted cells are lost through immunological clearance 13, 35. Our congenic transplantation showed rapid engraftment loss over 24 weeks after initial chimerism of 2.3–4.9% at a comparable dose of 5E+6 cells/fetus; this shortfall in long-term stability is probably related to other intrinsic barriers in this disease model. Human FL cells have demonstrated distinct competitive advantages over adult BM and UCB HSC in transplantation and a postnatal dose of 1E+6 resulted in partial erythropoietic correction in a neonatal thalassaemic murine model 42, 43. The chimerism of 1.8–2.4% that we attained with FL-IUHCT were at the lower range reported by Hayashi et al. of 2–10% with 1E+6 FL cells/fetus, which improved hematological indices transiently [33]. There are practical and ethical challenges of using FL in the presence of suitable alternatives such as maternal BM and UCB HSC 17, 44. FL-IUHCT in human recipients and large animals has, in contrast to murine and ovine studies 33, 45, consistently produced low and transient chimerism 11, 15 and similarly poor engraftment in postnatal recipients [46]. CD34 harvest from FL or UCB is limited, although the typical yield will be sufficient for fetal transplantation [47]. Maternal BM is readily available for repeat transplantations and produced 22% chimerism after intracardiac delivery of 2.5-5.2E+10 cells/kg at 0.67 G in canines [17], though our own experience with maternal BM-IUHCT in macaques using ~1E+9 cells/kg in early gestation has produced low macrochimerism and microchimerism (unpublished data).

Transfusion independence in successfully transplanted patients with thalassemia requires 10–20% engraftment of normal HSCs, facilitated by aggressive myeloablation aimed at maximal reduction of BM cells because persistent engraftment depends heavily on minimizing residual host cells 48, 49. With IUHCT in the thalassemic mouse, without the benefit of BM clearance, a single dose of 5E+6 adult BM donor cells/fetus resulted in short-term chimerism in our study (2.3–4.9%) similar to other β-thalassemia murine studies (1.1–4.1%) using the same dose 33, 38. Chronic changes in the BM microenvironment impair the capacity of transduced HSCs to engraft and mature in the long term [16]. The thalassemic niche is under constant physiological stress from ineffective erythropoiesis and compensatory expansion of erythroid progenitors; osteoporosis and osteopenia may add further strain [50]. These events alter the macromolecular structure and biochemical content of BM cells affecting interactions between HSCs and other BM cells, which may contribute to lower engraftment efficacy 51, 52. IUHCT-treated β-thalassemia mice illustrate this, with fivefold lower chimerism than WT pups treated with the same donor cell dose 33, 36, 38, as does IUHCT in human fetuses diagnosed prenatally with major hemoglobinopathies [15].

IUHCT may be most useful as part of a multipronged approach to induce donor-specific tolerance in utero before postnatal transplantation to maintain therapeutic engraftment. These combined approaches have employed booster doses of 30E+6 cells/pup, which resulted in >1% chimerism mainly due to the competitive advantage conferred by BM clearance or CD26 inhibition preceding transplantation 25, 28, 38, 53. High-dose total body irradiation (TBI) improved the initial ~2% chimerism in thalassaemic mice to ~70% (vs. ~ 15% without TBI), which was sufficient to correct splenomegaly and erythropoiesis 38, 53. Enhanced postnatal engraftment was similarly achieved with CD26 inhibition or pretransplantation high-dose busulfan 25, 28. We used IP-IUHCT of 5E+6 cells/fetus to create donor cell tolerance, followed by busulfan and fludarabine to create space and maintain peripheral tolerance to postnatal boost. Fludarabine is well tolerated when combined with busulfan in reduced-toxicity conditioning regimens for allogenic HSC transplantation for a range of hematological diseases [54]. We also used a much lower booster dose of 10E+6 cells/pup at 4 weeks (~20 g) and would have delivered 5E+8 cells/kg, similar to the optimal nucleated cell dose for postnatal BM transplantation in juveniles of 4E+8 cells/kg [55]. We were unable to consistently provide sufficient donor cells for doses of 30E+6 cells/pup. Therefore, we increased postnatal immunosuppression while keeping the booster dose low to determine our ability to maintain chimerism >1.8%, the threshold associated with sustained engraftment and tolerance in mice [14]. We observed a lower chimerism of 3–20% among boosted chimeric animals. Treated HET mice still showed improved MCV and RDW and smaller spleens compared with untreated HET mice. Although Hb was still lower than WT mice, the differences in MCV and MCH between treated HET and WT mice were now insignificant, similar to other studies [33]. Because perinatal TBI carries significant toxicity [56], interventions to overcome host competition have been reviewed, including early high-dose transplantation before endogenous BM population [17] and host BM clearance with anti-c-kit receptor antibodies [57]. However, to achieve IUHCT before BM population in a human fetus, prenatal diagnosis of a major hemoglobinopathy should be completed before 16 weeks' gestation because fetal BM erythropoiesis begins at 16–18 weeks (0.4–0.45 G) [58]. Although possible, we anticipate that the majority of at-risk patients will be diagnosed after this gestation and some degree of BM clearance will still be necessary to boost engraftment.

GLOBE-IUGT significantly improved hematological indices in treated HET mice, although with incomplete phenotype correction. This is the second demonstration of a therapeutic effect with direct LV-IUGT in thalassaemic mice [20]. Ex vivo gene transfer in juveniles and adults with β-hemoglobinopathies demonstrate varying degrees of success, from modest hemoglobin improvement to complete transfusion independence [21]. Hemopoietin correction and oncogenic risk both increase with higher VCN 24, 59, 60. Erythropoeitic correction is anticipated with LV-mediated HSC transduction of ~10–20% and severe murine β-thalassemia intermedia was cured with a VCN of 1–2.5 LV copies/HSC 24, 59, 60. We used a LV dose of 5E+6 TU/mL, 1–2 log-folds lower than doses in published studies, to keep VCN to <1 copy/nucleated blood cell, which carries the lowest risk of integration mutagenesis 59, 60, 61. At this low VCN, we still observed a significant improvement in Hb, MCV, and RBC counts and a trend toward a lower RDW in treated compared with untreated HET mice. Although these values were significantly different from WT mice, they suggest the utility of in vivo LV-IUGT once dosage is optimized. Improved design and enhanced therapeutic efficacy expand the utility of LV-mediated HSC transduction for affected individuals for whom curative HSC transplantation is unavailable or prohibitively expensive [62]. Of particular concern is the increased integration potential near growth control regions due to the open structure of fetal chromatin [63]. GLOBE has a low capacity for genotoxicity because of low-frequency integration, transgene expression restricted to differentiated erythropoietic cells, and low incidence of aberrant gene splicing in human cell lines 24, 60 and this profile is unlikely to change with in utero administration.

Although both direct LV-IUGT and IUHCT are potential options for fetal therapy, consensus opinion is that IUHCT is more suitable for clinical trials presently due to unanswered questions regarding IUGT safety; however, recent promising clinical trials in children may soon change this perspective 64, 65. It may be argued that intrauterine therapy should be reserved for historically lethal α-thalassemia major. With the considerable limitations of conventional therapy, fetal intervention will still provide benefit for the chronic β-hemoglobinopathies. IUHCT will be valuable in a multipronged strategy aimed at donor cell tolerance through fetal chimerism and enhanced engraftment through postnatal therapy.

## Conflict of interest disclosure

The authors declare no competing financial interests.

## Supplementary data

Appendix S1 Experimental Hematology Supplemental Data. Appendix S1
