# Supplementary material for: A comparison of intrauterine hemopoietic cell transplantation and lentiviral gene transfer for the correction of severe β-thalassemia in a HbbTh3/+ murine model
Source: Exp Hematol. 2018 Jun;62:45–55. doi: 10.1016/j.exphem.2018.03.006 (PMC5965454; doi:10.1016/j.exphem.2018.03.006)
Supplement: Appendix S1 — Experimental Hematology Supplemental Data. [file mmc1.pdf]

EXPERIMENTAL HEMATOLOGY SUPPLEMENTAL DATA

Ref.: Ms. No. 17- 140R1

A comparison of intrauterine haemopoietic cell transplantation and lentiviral gene transfer for the correction of severe <beta>-thalassaemia in the HbbTh3/+ murine model.

METHODS

Animal breeding

The breeding of HbbTh3/+ females and males was intended to generate homozygous fetuses in an attempt to rescue the β-thalassaemia major (β^0/β^0) phenotype through the proposed cell and gene therapy interventions (Figure 1A(i)). However, this resulted in small litters with low perinatal survival particularly after intravenous injections of GLOBE although we endeavoured to minimise the duration of surgical stress. Cross-breeding B6 females with HbbTh3/+ males resulted in surviving pups that were wild-type B6 (WT) or HbbTh3/+ in the ratio of approximately 4:1 (Figure 1Aii). Cross-breeding Crl:CD1(ICR) females (CD1®, Charles River Laboratories, Wilmington, MA) with HbbTh3/+ males resulted in hybrid pups heterozygous for HbbTh3/+ (HET) with better perinatal survival (Figure 1Aiii). Perinatal mortality was addressed by cross-fostering new pups with parturient CD1 dams as previously described [26].

Genotype of murine offspring

Offspring were assessed as heterozygous hybrid pups (HET), heterozygous HbbTh3/+ or WT by peripheral blood smear analysis showing defective erythropoiesis and genotypes were confirmed by reverse-transcriptase PCR [1, 2]. Genomic DNA extracted from tail-clips of 4-week old pups was processed using DNeasy Blood and Tissue kit (Qiagen, Hilden, Germany) according to manufacturer’s instructions. 2µl of eluted DNA was used to amplify a 249bp segment of the mouse β-major globin gene (*Hbb-b1*) with forward primer 5’-

TGAGAAGGCTGCTGTCTCTTG-3’ and reverse primer 5’-CAGAGGATAGGTCTCCAAAGCTA-3’. Equivalent loading was assessed by amplifying a 315bp segment of the *Hprt* housekeeping gene with forward primer 5’-GATGGGAGGCCATCACATTGTAG-3’ and reverse primer 5’-GCGACCTTGACCATCTTTGGATTA-3’. Amplification was performed over 40 cycles of 94°C for 30s, 55°C for 30s, 72°C for 2min and extension at 72°C for 7 minutes.

Postnatal donor cell transplantation

Postnatal transplantations were performed at 4-16 weeks in IUHCT-treated pups with B6-GFP donor cells. Cell recovery was 70-80% and viability ~70% following Ficoll processing and cryopreservation, similar to previous reports [35]. All mice selected for postnatal transplantation first underwent busulphan (Orphan Medical, MN, USA) conditioning with 35mg/kg [3] and a sub-group were given fludarabine (Sanofi, Surrey, UK) prepared by dissolving 5mg in 1ml dimethylsulfoxide (DMSO; Sigma); 100 µL was mixed with 900 µL of sterile water for a 0.5 mg/ml working solution and injected IP daily over two days. On day 3 busulfan was administered IP followed by IV tail-vein injection of CD26-inhibited B6-GFP cells in 0.5mL on day 4.

RESULTS

Generation of the murine intrauterine model system with surrogacy and surveillance

Hybrid HET pups displayed the characteristics of β-thalassaemia intermedia, being pale at birth (Figure 1B) with peripheral blood smears (PBS) displaying microcytosis and anisocytosis (Figure 1C). HET offspring had splenomegaly (Figure 1D), amplified murine *Hbb-b1* and human *Hprt*1 gene products on PCR analysis (Figure 1E) and had altered haematological indices compared with WT animals including lower haemoglobin (Hb) levels (8.9 ±1.4g/dL vs. 12.8 ±4.0g/dL, p=0.001), lower red blood cell (RBC) counts (6.4 ±2.0 $x10^6$ vs. 11.6 ±2.5 $x10^6$, p<0.001), higher reticulocyte distribution width (RDW, 39.1 ±1.2µm vs. 18.2 ±2.40µm, p<0.001) (Figure 1F).

FIGURE LEGEND

Figure S1: Murine model of major β-thalassaemia in the HbbTh3/+ mouse. (A) This model was established by mating HbbTh3/+ males and females to generate β^0/β^0 homozygote pups but only heterozygote offspring survived (i). Poor perinatal outcomes improved with breeding HbbTh3/+ males to WT females (ii) or to CCR2 or CD1 females resulting in hybrid offspring bearing the thalassaemic phenotype (HET, iii). To further improve perinatal survival pups were immediately fostered to co-parturient CD1 females. HET pups, like HbbTh3/+ heterozygotes, were pale (B), showed microcytosis and anisocytosis on peripheral blood smear (C, 4X magnification) and splenomegaly (D), and amplified murine *Hbb-b1* and human *Hprt*1 gene products on PCR (D). (F) HET displayed significantly lower haemoglobin (Hb) and red blood cell (RBC) counts, and higher red cell distribution width (RDW), than WT.

Figure S1

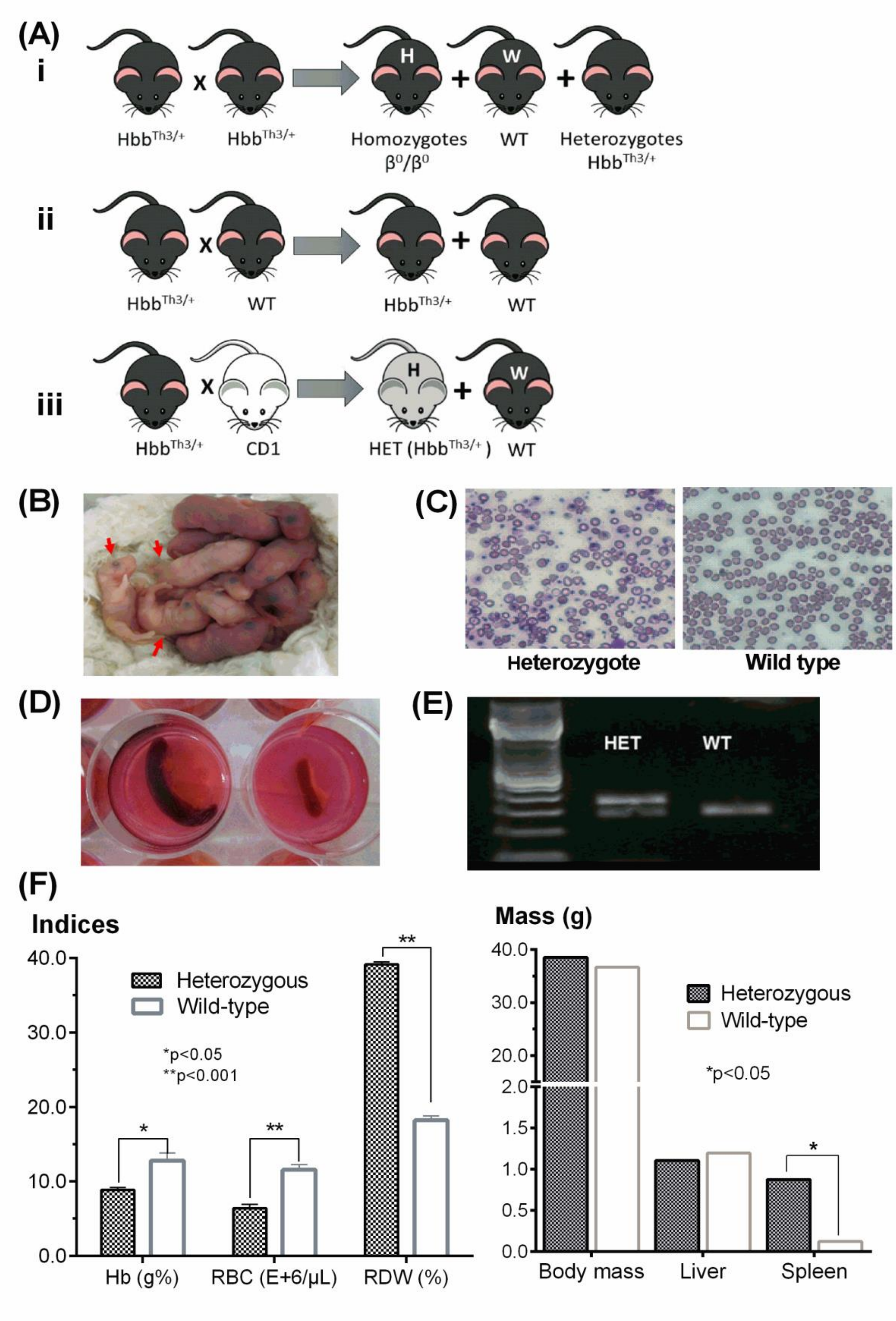
(A)
i
H
W
Hbb^Th3/+
Hbb^Th3/+
Homozygotes β0/β0
WT
Heterozygotes Hbb^Th3/+
ii
Hbb^Th3/+
WT
Hbb^Th3/+
WT
iii
Hbb^Th3/+
CD1
HET (Hbb^Th3/+)
WT
(B)
(C)
Heterozygote
Wild type
(D)
(E)
HET
WT
(F)
Indices
Heterozygous
Wild-type
*p<0.05
**p<0.001
40.0
30.0
20.0
10.0
0.0
Hb (g%)
RBC (E+6/μL)
RDW (%)
Mass (g)
Heterozygous
Wild-type
*p<0.05
40.0
30.0
20.0
2.0
1.5
1.0
0.5
0.0
Body mass
Liver
Spleen

[1] Yang B, Kirby S, Lewis J, Detloff PJ, Maeda N, Smithies O. A mouse model for beta 0-thalassemia. Proc Natl Acad Sci U S A. 1995;92:11608-11612.

[2] Ciavatta DJ, Ryan TM, Farmer SC, Townes TM. Mouse model of human beta zero thalassemia: targeted deletion of the mouse beta maj- and beta min-globin genes in embryonic stem cells. Proc Natl Acad Sci U S A. 1995;92:9259-9263.

[3] Ashizuka S, Peranteau WH, Hayashi S, Flake AW. Busulfan-conditioned bone marrow transplantation results in high-level allogeneic chimerism in mice made tolerant by in utero hematopoietic cell transplantation. Exp Hematol. 2006;34:359-368.
